# Fibrosis in Arrhythmogenic Cardiomyopathy: The Phantom Thread in the Fibro-Adipose Tissue

**DOI:** 10.3389/fphys.2020.00279

**Published:** 2020-04-03

**Authors:** Angela Serena Maione, Chiara Assunta Pilato, Michela Casella, Alessio Gasperetti, Ilaria Stadiotti, Giulio Pompilio, Elena Sommariva

**Affiliations:** ^1^Vascular Biology and Regenerative Medicine Unit, Centro Cardiologico Monzino IRCCS, Milan, Italy; ^2^Heart Rhythm Center, Centro Cardiologico Monzino IRCCS, Milan, Italy; ^3^University Heart Center, Zurich University Hospital, Zurich, Switzerland; ^4^Department of Clinical Sciences and Community Health, University of Milan, Milan, Italy

**Keywords:** arrhythmogenic cardiomyopathy, cardiac fibrosis, cardiac extracellular matrix, scar formation, cellular effectors

## Abstract

Arrhythmogenic cardiomyopathy (ACM) is an inherited heart disorder, predisposing to malignant ventricular arrhythmias leading to sudden cardiac death, particularly in young and athletic patients. Pathological features include a progressive loss of myocardium with fibrous or fibro-fatty substitution. During the last few decades, different clinical aspects of ACM have been well investigated but still little is known about the molecular mechanisms that underlie ACM pathogenesis, leading to these phenotypes. In about 50% of ACM patients, a genetic mutation, predominantly in genes that encode for desmosomal proteins, has been identified. However, the mutation-associated mechanisms, causing the observed cardiac phenotype are not always clear. Until now, the attention has been principally focused on the study of molecular mechanisms that lead to a prominent myocardium adipose substitution, an uncommon marker for a cardiac disease, thus often recognized as hallmark of ACM. Nonetheless, based on Task Force Criteria for the diagnosis of ACM, cardiomyocytes death associated with fibrous replacement of the ventricular free wall must be considered the main tissue feature in ACM patients. For this reason, it urges to investigate ACM cardiac fibrosis. In this review, we give an overview on the cellular effectors, possible triggers, and molecular mechanisms that could be responsible for the ventricular fibrotic remodeling in ACM patients.

## Introduction

Arrhythmogenic cardiomyopathy (ACM) is a rare genetic cardiac disease, with an incidence estimated in 1:5000 (Corrado et al., 2017), which affects predominantly the right ventricle (RV), although left or biventricular forms have been also described. In about 50% of ACM patients, a genetic mutation can be identified, mostly in genes coding for cardiac desmosomes. Non-desmosomal forms of ACM also exist. The mode of inheritance is generally autosomic dominant, even if recessive syndromic forms are also described (Stadiotti et al., 2019). However, these different genetic determinants lead to a similar disease phenotype. All forms of ACM are characterized by incomplete penetrance and variable expressivity even in carriers of the same causative mutation. This characteristic likely means that different factors, such as genetic background, or environmental determinants, contribute to define the clinical phenotype. From an anatomo-pathological point of view, ACM hearts show a progressive loss of myocardium, inflammatory infiltrates, and fibrous or fibro-fatty replacement. Such tissue heterogeneity predisposes to re-entrant electrical activity that is known to support ventricular arrhythmias, cause, in the worst-case scenario, of sudden cardiac death. Specifically, cardiac fibrosis is originally a protective mechanism against injury, but its uncontrolled progression may lead to excessive collagen deposition and myocardial scar formation. The fibrotic molecular mechanisms are known for cardiac diseases but those specific for ACM still need to be investigated in order to uncover therapeutic targets to improve ACM clinical management.

## Clinical Aspects of Fibrosis in ACM Patients

Arrhythmogenic cardiomyopathy is a rare cardiac pathology characterized by cardiomyocytes (CM) death and replacement of myocardium with fibrotic or fibro-fatty tissue. The fibrotic and fibro-fatty substitution, regardless of the ventricular district, progresses from epicardium to endocardium provoking structural and functional myocardial alterations (Lin et al., 2018). Generally, segmental or irregular fibro-fatty tissue distribution can be observed among patches of CM (Hoorntje et al., 2017). In most cases, the region of the heart typically interested by pathological changes is the RV where abnormal myocardium remodeling is localized in the so-called “triangle of dysplasia,” composed by RV inflow tract, RV outflow tract, and RV apex. In particular, during the first stages of the disease, the basal inferior RV region is usually compromised, while RV apex involvement occurs in advanced phases of ACM progression (Te Riele et al., 2013). Fibrosis in the ventricular septum is rare (Hoorntje et al., 2017).

Although ACM has long been defined as a pathology of the RV, left ventricle (LV) involvement has also been reported either in advanced stages of the RV disease or in peculiar LV-dominant forms. Particularly, in the LV, myocardial remodeling mainly affects the posterolateral area and the original concept of “triangle of dysplasia,” has evolved to a new scenery of a “quadrangle of ACM” (Te Riele et al., 2013). It has been shown that the LV involvement is different based on the genetic defect. Specifically, more fibrosis is located in the LV free wall of the ACM hearts mutated for phospholamban (*PLN*) than those mutated in desmosomes (Sepehrkhouy et al., 2017). Among desmosomal gene mutations, desmoplakin (*DSP*) or desmoglein 2 (*DSG2*) are often associated with LV-forms (Norman et al., 2005; Pilichou et al., 2006; Sen-Chowdhry et al., 2008). Mechanisms of regional-differences are still to be investigated.

The presence of fibro-fatty substitution in ACM hearts could be evidenced through different diagnostic tools. Echocardiography is the standard imaging technique used to evaluate structural and functional abnormalities of the RV chamber, although it provides limited information on the presence and extent of fibrotic replacement. For these reasons, magnetic resonance imaging (MRI) is currently increasingly recommended for a definitive diagnosis (de Boer et al., 2019). MRI allows to asses ventricular volumes, systolic function, and regional wall motion that are included in diagnostic criteria (Marcus et al., 1982, 2010). Moreover, MRI can detect adipose tissue, and, thanks to gadolinium delayed contrast enhancement acquisition, MRI is the gold-standard exam to characterize myocardial tissue in terms of fibrosis, fatty infiltration, and fibrofatty scar (Kim et al., 1999, 2000; Tandri et al., 2002, 2005).

Invasive tissue characterization to confirm ACM diagnosis can be obtained by an endomyocardial biopsy (EMB). In this setting, an extensive application of EMB has been limited by the low sensitivity of biopsies usually obtained from the interventricular septum, not frequently involved by the disease. In the last few years, EMB is performed after a complete and detailed 3D electroanatomic mapping (EAM) of the ventricular chamber. EAM is used to detect bipolar and/or unipolar low potential areas, which, in ACM, mostly correspond to fibrotic or fibro-adipogenic scar tissue (Santangeli et al., 2012; Casella et al., 2015). Thus, a preliminary EAM allows to directly identify fibrotic substitution areas and perform EMB in the portion of RV wall in the immediate adjacency of the scar (Casella et al., 2017). However, EAM and EAM-guided EMB are not yet recognized in task force guidelines (Santangeli et al., 2011).

## Pro-Fibrotic Triggers

The compromised heart of ACM patients undergoes a functional worsening when subject to intense physical exercise. The practice of physical activity at a competitive level represents one of the major triggers for life-threatening arrhythmias and sudden death in the ACM setting and therefore it is highly discouraged for ACM patients (Cerrone, 2018). Endurance athletes become symptomatic at an earlier age, more likely develop an overt phenotype, and show more frequently ventricular arrhythmias and heart failure (James et al., 2013). Therefore, an athletic lifestyle affects disease penetrance. Furthermore, it is associated with the activation of the sympathetic nervous system, mechanical and oxidative stress, which may prime ACM pathogenesis.

It has been reported that sympathetic dysinnervation characterize both the left (Wichter et al., 1994; Paul et al., 2011) and the RVs (Todica et al., 2018) of ACM patients: the areas affected by myocardial replacement show reduced reuptake of norepinephrine leading to chronic stimulation of adrenergic receptors, which in turn has been related to cardiac fibrosis. The norepinephrine treatment induces cardiac fibrosis promoting a series of events, such as CM death and collagen and transforming growth factor beta 1 (TGFβ1) gene expression in rats’ ventricular endocardium (Bhambi and Eghbali, 1991; Castaldi et al., 2014). TGFβ1 overexpression, in turn, could promote an increase of β-adrenergic expression, further enhancing interstitial fibrosis (Iizuka et al., 1994; Mak et al., 2000; Rosenkranz et al., 2002).

Moreover, the β-adrenergic system could regulate the extracellular matrix (ECM) protein turnover: norepinephrine could increase the expression of metalloproteinase-2 (MMP-2) and decrease the expression of tissue inhibitors of metalloproteinases 1 and 2 (TIMP-1/2) during cardiac remodeling (Briest et al., 2001; Meier et al., 2007).

High-level sport activity also implies an excessive effort of the heart muscle.

Excessive heart stimulation can cause mechanical stretch of fibers. It has been reported that athletes with a history of endurance sport have increased levels of plasmatic TGFβ1 and develop myocardial fibrosis in contrast to novice athletes (Heinemeier et al., 2003; Czarkowska-Paczek et al., 2006). While the physiological adaptation to strength training causes a pressure load and resulting eccentric hypertrophy, endurance exercise causes a volume load and ventricular dilation mostly affecting the RV (Morganroth et al., 1975; Wilson et al., 1985; La Gerche et al., 2012). Interestingly, a positive loop promoting fibrosis is described: changes in ECM composition during cardiac fibrosis alter the mechanical tissue properties increasing its rigidity. Tissue stiffness further promotes the differentiation of myofibroblasts that produce and release collagen. Collagen deposition, in turn, increases stiffness of the tissue (Hinz, 2009). Independently of ACM, exercise training is a known source of fibrotic cardiac remodeling. A rat model of intensive training is characterized by increased cardiac mass, diffuse interstitial collagen deposition, and increased levels of TGFβ1, fibronectin-1, and MMP-2. Intriguingly, detraining can revert the cardiac remodeling observed to control levels (Benito et al., 2011).

In ACM patients, due to (Wang et al., 2018) the genetically determined fragility of desmosomes, the mechanical stretch of CM during endurance exercise may favor cell injury and accentuate the development of the disease. Moreover, a mechanotransduction mechanism (the Hippo pathway), translating mechanical stimuli into activation of fibro-adipogenic signals, is known to participate in ACM pathogenesis (Dupont et al., 2011; Chen et al., 2014). Cardiac overload reduction therapies have been proposed based on ACM animal model findings (Fabritz et al., 2011). Detraining has only a limited effect on arrhythmias reduction (Wang et al., 2018).

Excessive training is also associated with oxidative stress increase (Fabritz et al., 2011; Wang et al., 2018). Uncontrolled reactive oxygen species (ROS) balance causes cell necrosis and apoptosis due to ROS oxidizing effects on proteins, lipids, and DNA, and prompting of pathway modifications (Moris et al., 2017). ROS are involved in the development and progression of cardiovascular diseases, such as cardiac hypertrophy, heart failure, and hypertension (Rani et al., 2016; Siasos et al., 2018). Moreover, oxidative stress is linked to cardiac fibrotic remodeling by regulating fibroblast function and ECM composition. TGFβ1 and ROS positively affect each other during myofibroblast differentiation. Particularly, TGFβ increases oxidative stress by inducing ROS production by mitochondria and decreasing the activity of antioxidant enzymes (Purnomo et al., 2013; Liu and Desai, 2015). In particular, TGFβ1 acts: (1) on mitochondrial ROS production by inducing the expression of NAD(P)H Oxidases4; (2) reducing the concentration of glutathione. Both these events typically occur in fibrotic disease (Cucoranu et al., 2005; Liu and Gaston Pravia, 2010). On the other hand, ROS promote the generation of active TGFβ and regulate ECM protein expression and degradation acting on synthesis and activity of MMPs (Barcellos-Hoff and Dix, 1996; Siwik et al., 2001; Jacob-Ferreira and Schulz, 2013).

Although numerous pieces of evidence concur to a role of oxidative stress in fibrosis, its implication in ACM fibrotic remodeling still to be investigated. Indeed, only one report described increased ROS levels in an ACM cell model (Kim et al., 2013).

An important independent fibrosis cofactor in ACM hearts is inflammation. ACM hearts are characterized by progressive CM death that is replaced by non-contractile fibrotic tissue according to a reparative mechanism against myocardial loss (Valente et al., 1998; Rusciano et al., 2019).

Cardiac fibroblasts and CM are in contact through soluble factors and cell–cell interactions. CM death may represent the initial phase in the remodeling process, by initiating an inflammatory response, myofibroblast activation, and myocardial scar formation (Frangogiannis, 2008; Kakkar and Lee, 2010; Suthahar et al., 2017). Moreover, during inflammation, inflammatory cytokines IL-6, TNFα, and IL-1β are upregulated and involved in promoting cardiac fibroblast proliferation and activation (Plenz et al., 1998; Ferrari, 1999; Turner et al., 2007; Bujak and Frangogiannis, 2009).

Transgenic mice with cardiac restricted overexpression of TNFα exhibit increased collagen synthesis and deposition, MMP-2 and MMP-9 activity and TGFβ expression (Sivasubramanian et al., 2001). Furthermore, it has been demonstrated that the suppression of the IL-1 signaling ameliorates the adverse fibrotic remodeling in association with a reduced inflammation (Bujak et al., 2008). The presence of inflammatory cell patches, mostly macrophages, neutrophils, and T-lymphocytes, in the ventricular wall affected by CM death, has been reported in ACM heart along with a high plasmatic level of pro-inflammatory cytokines (Campian et al., 2010; Asimaki et al., 2011; Campuzano et al., 2012). It has been observed that NFκB signaling is activated in ACM mouse and cell models characterized by different causative desmosomal gene variants. The inhibition of NFκB signaling is able to rescue, *in vitro*, different ACM phenotypic features as distribution of plakoglobin (PG), Cx43, and GSK3β, apoptotic rate, and inflammatory cytokines production. *In vivo*, the pharmacological inhibition of NFκB signaling improves contractile function, reduces the amount of ventricular myocardial necrosis and fibrosis and the number of apoptotic cells, and normalizes the ECG abnormalities (Chelko et al., 2019). This evidence hints to a primary role of inflammation in ACM. In a translational prospect, targeting inflammation could improve different aspects of ACM pathogenesis.

Arrhythmogenic cardiomyopathy most frequently occurs in men, with more severe clinical complications compared to women (Bauce et al., 2008). ACM affected women are characterized by low serum levels of estradiol and raised cardiovascular events underling the cardioprotective role of this hormone. In contrast, a high level of testosterone has been found in the ACM male serum, in line with previous data describing the involvement of testosterone in arrhythmia induction (Ayaz and Howlett, 2015; Akdis et al., 2017; Stadiotti et al., 2019).

Interestingly, the development of cardiac fibrosis has also been linked to gender-associated differences. During cardiac fibrosis collagen type I and III deposition is higher in men compared to women (Kararigas et al., 2014; Regitz-Zagrosek and Kararigas, 2017).

The molecular mechanisms underlying the cardioprotective role of estrogens have not been fully clarified (Piro et al., 2010). It is known that female hormones inhibit cardiac fibroblast proliferation and their capability to synthesize and deposit collagen (Dubey et al., 1998).

Notably, the estradiol differentially acts on collagen expression in cardiac fibroblasts in a gender-dependent manner. Indeed, an estradiol treatment decreases collagen I and III expression in female derived cardiac fibroblasts *via* estradiol receptor α, while in men cardiac fibroblasts, the activation of estradiol receptor β induces the upregulation of collagen synthesis (Mahmoodzadeh et al., 2010).

Moreover, the estradiol could regulate ECM turnover by affecting the expression of MMP-2, which in turn is associated with altered ventricular remodeling in different cardiovascular pathologies (Dworatzek et al., 2019).

The anti-fibrotic effects of estradiol have also been reported in a mouse model of heart failure where the treatment reduces the expression of TGFβ1 and profibrotic genes, like collagen I, and therefore suppresses cardiac fibrosis (Iorga et al., 2016). One report demonstrated the role of sex hormones on different ACM phenotypes in an ACM CM model (Akdis et al., 2017). Nevertheless, further investigations are needed in order to link the sex hormones involvement to ACM associated fibrosis.

## Cardiac Extracellular Matrix Regulation

The excessive deposition of fibrous connective tissue leads to the formation of a myocardial scar which contributes to the dysregulation of cardiac electrical properties and thus to arrhythmic events.

Cardiac ECM is a well-organized network composed of support proteins that create a solid substrate in which myocytes and non-contractile cells such as fibroblasts, leukocytes, and endothelial cells are placed (Aggeli et al., 2012).

The cardiac ECM supporting fibers are predominantly composed of collagen type I (which forms thick fibers that ensure tensile strength), collagen type III (which forms thin fibers that ensure elasticity) and in a minor fraction by collagen type IV, V, and VI. Moreover, cardiac ECM contains glycosaminoglycans, glycoproteins, and proteoglycans (Frangogiannis, 2012). The ECM also plays a non-structural function supplying growth factors, cytokines, and proteases necessary for cardiac function, cardiac cell destiny, and homeostatic regulation (Rienks et al., 2014).

Extracellular matrix deposition is mostly associated with fibroblasts activation. Different proteinases such as matrix MMPs and TIMPs overall act to a fine regulated homeostatic balance between synthesis and degradation (Kassiri and Khokha, 2005; Spinale et al., 2016).

Following cardiac injury, ECM degradation occurs and promotes inflammatory cell infiltration and fibroblast proliferation. The following fibroblasts to myofibroblasts differentiation represents the event responsible for consistent novel ECM deposition during scar formation.

Alterations in ECM composition and turnover are involved in different cardiac diseases characterized by adverse remodeling with loss of myocardium integrity (Swynghedauw, 1999; Aggeli et al., 2012; Santulli et al., 2012; Cipolletta et al., 2015). Patients affected by idiopathic dilated cardiomyopathy are characterized by an excessive deposition of collagen type III fibers that are poorly cross-linked and lead to cell slippage, ventricular dilatation, and altered diastolic compliance (Gunja-Smith et al., 1996). Furthermore, altered expression of TIMP and MMP levels have been found in the explanted hearts of these patients while increased plasma concentrations have been associated with systolic dysfunction during hypertrophic cardiomyopathy (Brilla et al., 1994; Tyagi et al., 1996; Thomas et al., 1998; Noji et al., 2004).

The molecular basis of ECM organization and remodeling in ACM is still under-investigated. Recently few papers identified a signature of ACM cardiac cell microRNAs, known to be involved in ECM turnover and mechanosensing (Rainer et al., 2018; Puzzi et al., 2019).

## Cellular Effectors

Cardiac injury represents a trigger for the activation of immune cells that in turn stimulate fibroblasts proliferation and differentiation in myofibroblasts. During physiological cardiac repair, after the wound closure, myofibroblasts apoptosis occurs with consequent resolution of the process. On the contrary, during pathological conditions, myofibroblast secretory activity results extended, inducing the switch from reparative process to fibrotic scar formation (Tomasek et al., 2002; Santiago et al., 2010; Stempien-Otero et al., 2016; Murtha et al., 2017).

To date, the cellular source of myofibroblasts is still not fully defined. The most reliable hypothesis is that resident cardiac fibroblasts are activated during damage, as following pressure overload, with consequent differentiation into myofibroblasts. Notably, it has been reported that ventricular resident Tcf21 positive fibroblasts are a source of myofibroblasts involved in cardiac fibrosis after myocardial infarction (Moore-Morris et al., 2014; Furtado et al., 2016; Kanisicak et al., 2016).

In this context, it is known that epicardial cells undergo epithelial-to-mesenchymal transition (EMT) to generated fibroblasts that could populate the cardiac injury area promoting fibrotic remodeling (Russell et al., 2011; Ruiz-Villalba et al., 2013). Notably, typical pro-fibrotic factors such as TGFβ can induce the EMT of the epicardial cells after cardiac injury (Zeisberg et al., 2007).

Recently, a subset of resident adult cardiac stem cells characterized by the expression of PW1 has been identified as responsible for fibrosis after myocardial infarction. The amount of PW1 positive cells is increased in the ischemic damaged area. PW1 cells are characterized by the high expression of profibrotic genes and the ability to differentiate into fibroblasts (Yaniz-Galende et al., 2017).

However, other studies indicate that cardiac fibroblasts could derive from resident cardiac mesenchymal cells (C-MSC). In the injured mouse heart, as during myocardial infarction, C-MSC resident population (not recruited from the bone marrow) express stem cell and fibroblast markers like collagen type I and DDR2, suggesting their involvement in scar formation (Carlson et al., 2011). C-MSC have been involved as major player of ACM adipogenesis (Sommariva et al., 2016; Pilato et al., 2018). A C-MSC population isolated based on PDGFRα and Sca1 could be responsible for fibrofatty scar formation in ACM patients. In human and mouse hearts, the fibro-adipogenic progenitors (FAP) population have been implicated in the fibro-fatty substitution in ACM. Indeed, they were characterized as bi-potential cells, most with fibrous commitment, and a small percentage with fat genes expression. In particular, the cardiac FAP limited deletion of DSP leads to an increase interstitial fibrosis with a high TGFβ1 level in mice ventricular myocardium (Lombardi et al., 2016; Sommariva et al., 2017).

Moreover, the possible origin of cardiac fibroblasts from non-cardiac departments is still a matter of debate. It has been reported that bone marrow-derived cells could generate fibroblasts that are in turn involved in cardiac scar formation after myocardial infarction (van Amerongen et al., 2008). Indeed, EGFP positive cells, that are able to produce collagen I contributing to scar formation, have been found in the infarcted cardiac area of EGFP bone marrow chimeric mice. Bone marrow cells may represent the fibroblast population in the initial phase of the remodeling process but are not involved in the persistent fibrotic deposition (van Amerongen et al., 2008).

In addition, fibrocytes could be a further circulating source of cardiac fibroblasts as CD34/CD45 positive cells that expressed fibroblast markers and have been identified in a model of fibrotic ischemia/reperfusion cardiomyopathy (Abe et al., 2001; Haudek et al., 2006; Mollmann et al., 2006; Krenning et al., 2010).

## Molecular Mechanisms

The most well-known pro-fibrotic cytokine involved in cardiac fibrosis is TGFβ (Lloyd-Jones et al., 2009; Borthwick et al., 2013). It participates to tissue remodeling by: (1) promoting fibroblasts expansion and conversion into myofibroblasts; (2) inducing the production and deposition of ECM; and (3) preventing matrix degradation by increasing the expression of TIMP (Bujak and Frangogiannis, 2007).

Specifically, binding of TGFβ to its receptors is the starting point for the activation of downstream signaling cascade that involves different mediators of the canonical (SMADs proteins) or non-canonical (ERK, JNK, and p38 MAPK) pathways.

Although ACM is commonly defined as a “desmosomal disease” being the majority of the patients mutated in desmosomal genes, additional mutations have been identified in genes that encode for non-desmosomal proteins (Moccia et al., 2019). One of those in *TGFB 3*, responsible for the ARVD1 form (Beffagna et al., 2005). In ACM patients, mutations in *TGFB 3* are linked both to an increase in cardiac fibrotic remodeling and to the regulation of desmosomal gene expression (Beffagna et al., 2005; Tamargo, 2012). Interestingly, also the existence of a possible desmosomal protein-dependent TGFβ expression has been reported. Particularly, it has been demonstrated that plakophilin 2 (PKP2) and DSP control the activity of TGFβ1/p38 MAPK pathway both *in vitro* and *in vivo*. Indeed, in CM with a loss of PKP2, an increase in TGFβ1 signaling is observed with consequent fibrotic genes expression, like collagen and fibronectin (Li et al., 2011). Moreover, DSP expression is lost following *PKP2* knockdown. Since the restoration of DSP expression rescues the activation of TGFβ1/p38 signaling, DSP acts upstream TGFβ1/p38 and downstream PKP2 (Dubash et al., 2016).

Conversely, TGFβ1 treatment induces both an increase of DSP I and II expression and a reduction of DSP degradation in bronchial epithelia (Yoshida et al., 1992).

Overall, these observations demonstrate that TGFβ could modulate the expression of junctional proteins leading to the modification of cellular phenotype and promoting the formation of fibroblasts. In this context, it is important to underline that TGFβ promotes EMT, which is a process characterized by cell–cell contact changes (Zeisberg et al., 2007).

It has been hypothesized that, in ACM, desmosome mutations cause PG translocation from intercalated discs to the nucleus where it competes with β-catenin for the binding to TCF/LEF transcription factors based on the high structural homology (Garcia-Gras et al., 2006; Miravet et al., 2016). The abnormal PG translocation causes the altered canonical activation of Wnt/β-catenin signaling pathway promoting the pathological fibro-adipose myocardial tissue substitution (Garcia-Gras et al., 2006; Moccia et al., 2019).

Intriguingly, it has been reported that TGFβ influences Wnt/β-catenin signaling in a positive manner (Dzialo et al., 2018). TGFβ acts on canonical Wnt pathway in cardiac fibroblasts by: (1) inducing Wnt proteins release; (2) decreasing the expression of Wnt pathway inhibitors; and (3) inhibiting GSK-3β leading to the translocation of active β-catenin from the cytosol to the nucleus (Akhmetshina et al., 2012; Lal et al., 2014; Blyszczuk et al., 2017).

On the other hand, the action of Wnt1 ligand, overexpressed in ventricular epicardium after cardiac damage, causes the activation of Wnt pathway, with consequent differentiation of epicardial fibroblasts into myofibroblasts with collagen synthesis (Duan et al., 2012). The presence of Wnt ligands, in combination with decreased expression of Wnt pathway inhibitors, contributes to nuclear β-catenin localization in human fibroblasts during the fibrotic process while loss of β-catenin in cardiac fibroblasts reduced ECM gene expression and collagen deposition (Xiang et al., 2017).

It is important to emphasize that adipogenesis and fibrogenesis are differentiation programs well regulated by independent pathways. TGFβ1 induces myofibroblast differentiation reducing in parallel the expression of PPARγ, the mast regulator of adipogenic differentiation (Vallee et al., 2017). On the contrary, PPARγ acts preventing myofibroblasts differentiation and collagen deposition.

One further molecular mechanism involved in ACM pathogenesis as well as in myofibroblast differentiation is the Hippo pathway that acts by regulating YAP/TAZ shuttling between nucleus and cytoplasm. Specifically, in the ACM context, the altered PG distribution induces the retention of YAP into the cytoplasm with activation of Hippo pathway and suppression of canonical Wnt-related gene expression (Wada et al., 2011; Zhou and Zhao, 2018). Furthermore, during myofibroblasts differentiation, the YAP/TAZ nuclear localization is associated with Wnt activation and TGFβ1 increase level with consequent SMAD phosphorylation in fibrotic tissues (Liu et al., 2015, 2018; Piersma et al., 2015).

Recently, the activation of the adenosine 2A receptor (A2AR) has been reported to contribute to the progression of fibrosis in an ACM animal model (Cerrone et al., 2018). The binding of adenosine to A2AR stimulates expression of TGFβ, CTGF, and matrix production (Shaikh et al., 2016). Moreover, A2AR activation interacts with the Wnt pathway (Shaikh et al., 2016; Zhang et al., 2017).

## Conclusion

The ACM specific cardiac remodeling is characterized by the progressive substitution of ventricular myocardium of patients by non-contractile fibrotic or adipose tissue. While adipogenesis has been extensively studied in this pathological context, fibrosis, a cardiac phenotype common to most cardiac diseases, remains under-investigated.

**FIGURE 1:**
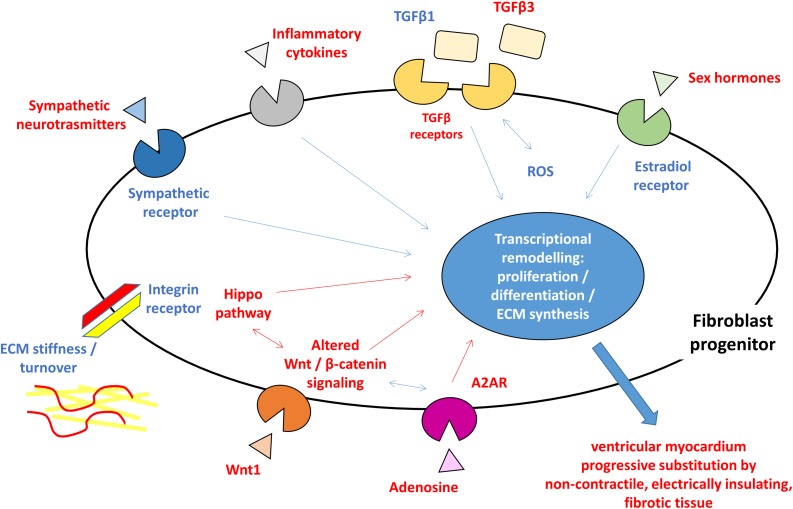
Schematic figure highlighting the hypothesized pro-fibrotic process in ACM. The presence of different triggers (sympathetic nervous system activity, extracellular matrix ECM component, reactive oxygen species ROS, inflammatory cytokines, and sex hormones) and the activation of molecular pathways (Hippo, Wnt/β-catenin, and TGFβ) lead to transcriptional rearrangement for excessive proliferation and myofibroblasts differentiation of fibroblast progenitors. These changes ultimately result in ventricular myocardium progressive substitution by non-contractile, electrically insulating, fibrotic tissue. In blue, what is known about pro-fibrotic mechanisms in general and hypothesized in ACM, in red what is reported for ACM pathogenesis. ACM: arrhythmogenic cardiomyopathy; A2AR: adenosine 2A receptor; ECM: extracellular matrix; ROS: reactive oxygen species; TGFβ: transforming growth factor β.

Myocardial fibrosis is a clinical feature shared by several heart diseases such as ischemic cardiomyopathy, dilated cardiomyopathy, hypertrophic cardiomyopathy, hypertensive heart disease, and heart failure. Ventricular fibrosis may develop different modality depending on disease progression and typically result in the formation of substrate vulnerable to arrhythmic events. The cardiac fibroblast activation and differentiation into myofibroblasts and the resulting scar formation commonly occur following a cardiac injury. This event represents a reparative process but during a pathological cardiac condition, it becomes a persistent status that leads to altered myocardial structure and function. As described in other cardiac diseases, the presence of fibroblasts and fibroblast progenitors, the excessive collagen deposition, and the following modification of mechanical stiffness may improve the tissue discontinuity occurring in the ACM hearts. Therefore, most of what is known about fibrotic processes and is summarized in this review is iterated from studies in other settings. However, it is expected that triggering agents, cellular effectors, and mechanisms are comparable to what previously described. Responsible cells are likely cardiac fibroblasts, either from FAP progenitors or C-MSC. ACM key pathogenic mechanisms such as Wnt and Hippo are playing direct roles, with the support of TGFβ-mediated mechanisms, which prompts fibrosis as an alternative to adipogenesis. The whole process is possibly triggered by genetically driven myocardial damage, and inflammation, oxidative stress, mechanical and neuro-hormonal signaling are magnifying factors (Figure 1), thus representing possible targets for therapies.

Nevertheless, ACM specific fibrosis remains a scientific gap of knowledge to be filled with further studies, in order to clarify specific pathways as target for novel specific therapeutic actions.

## Author Contributions

All authors contributed to the writing of the paragraphs and to the critical review of them.

## Conflict of Interest

The authors declare that the research was conducted in the absence of any commercial or financial relationships that could be construed as a potential conflict of interest.
